# Chronic hypoxia prolongs postoperative mechanical ventilation and reduces the left atrial pressure threshold in children with tetralogy of Fallot

**DOI:** 10.3389/fped.2022.965703

**Published:** 2023-01-06

**Authors:** Jiangshan Huang, Jie Ding, Xie Wu, Yuan Jia, Qiao Liu, Su Yuan, Fuxia Yan

**Affiliations:** Department of Anesthesiology, National Center for Cardiovascular Diseases, Fuwai Hospital, Chinese Academy of Medical Sciences and Peking Union Medical College, Beijing, China

**Keywords:** tetralogy of Fallot, child, oxygen saturation, hypoxia, left atrial pressure, mechanical ventilation

## Abstract

**Background:**

Chronic hypoxia induces pulmonary microvascular endothelial dysfunction. The left atrial pressure (LAP) represents the hydrostatic pressure of pulmonary microcirculation. The conjunction of the LAP and any abnormal pulmonary microvascular endothelial barrier function will have an impact on pulmonary exudation, resulting in prolonged mechanical ventilation. This study aimed to investigate the tolerance threshold of the pulmonary microcirculation to LAP in children with tetralogy of Fallot (TOF) to avoid prolonged mechanical ventilation after surgery.

**Methods:**

This retrospective study included 297 Chinese patients who underwent TOF correction at Fuwai Hospital. Patients were categorized according to their preoperative oxygen saturation (SpO_2_) level. One-to-one propensity score matching (PSM) revealed a total of 126 participants in the SpO_2 _< 90% and SpO_2 _≥ 90% groups. Between-group comparisons were conducted to verify the correlation between hypoxia and prolonged mechanical ventilation. A subgroup analysis was performed to reveal the significant role of postoperative LAP stewardship on prolonged mechanical ventilation.

**Results:**

Failure to extubate within the first 48 h (23.81% vs. 9.52%, *P* = 0.031) and prolonged mechanical ventilation (26.98% vs. 11.11%, *P* = 0.023) were more commonly observed in children with preoperative SpO_2_ < 90%. The incidence of prolonged mechanical ventilation consistently increased with LAP in both the SpO_2_ < 90% and SpO_2_ ≥ 90% groups, although LAP was still within the normal range (6–12 mmHg). Children in chronic hypoxic conditions tolerated lower LAP well. The tolerance threshold for postoperative LAP in children diagnosed with TOF under chronic hypoxic conditions was identified as 7 mmHg.

**Conclusions:**

Children in a chronic hypoxic state may suffer from a high incidence of prolonged mechanical ventilation after surgical correction of TOF and may not tolerate higher postoperative LAP. To improve pulmonary prognosis, it is better to control and maintain the postoperative LAP at a lower state (≤7 mmHg) in children with chronic hypoxia.

## Introduction

Tetralogy of Fallot (TOF) is a common cyanotic congenital heart disease. Due to pulmonary artery (PA) stenosis and chronic pulmonary bloodlessness, the lung compensates by growing collateral circulation to maintain the blood supply (1). The increased number of microcirculation vessels maintains lung function to some extent. However, weak neovascularization may explain pulmonary exudation is more likely to occur.

A large number of studies have shown that pulmonary microvascular endothelial dysfunction occurs under chronic hypoxia (2–4). Meanwhile, cardiopulmonary bypass (CPB) can further damage pulmonary microcirculation barrier function (5). Therefore, children with preoperative chronic hypoxia may be more prone to pulmonary exudation after surgical correction for TOF, resulting in prolonged mechanical ventilation (MV).

In the absence of significant differences in postoperative cardiac function and volume, left atrial pressure (LAP) essentially represents the hydrostatic pressure of the pulmonary microcirculation. Elevated LAP induces hemodynamic pulmonary congestion with high capillary pressure, which leads to increased fluid transfer to the capillary interstitial and alveolar space (6). This can result in edema formation and atelectasis with damaged gas exchange. We hypothesized that children with chronic hypoxia could only tolerate a lower level of postoperative LAP owing to impaired alveolar–capillary barrier function.

However, after a careful literature review, we could not find any clinical evidence of a higher incidence of related adverse pulmonary outcomes and specific LAP management in children with congenital heart disease and chronic hypoxia. Therefore, we retrospectively analyzed the relationship between preoperative oxygen saturation (SpO_2_) and postoperative prolonged mechanical ventilation in children undergoing TOF correction in our center and accurately confirmed the postoperative pulmonary LAP threshold in children with chronic hypoxia.

## Materials and methods

### Clinical study design

**Figure 1:**
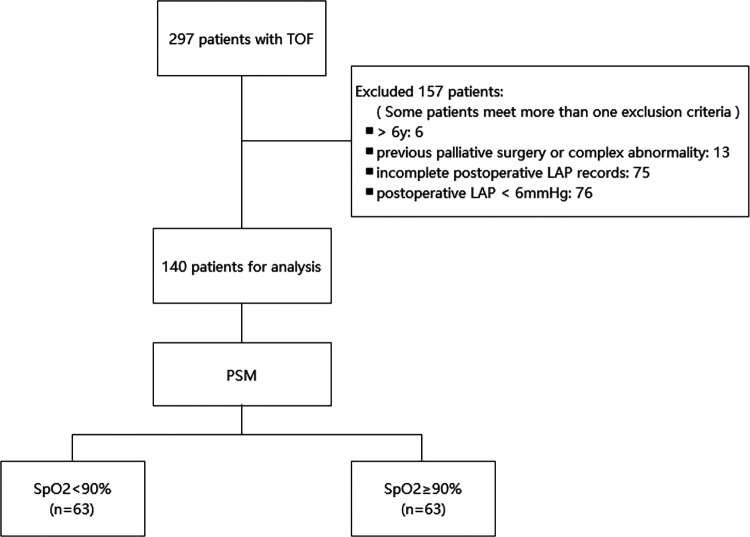
Flowchart of the study. LAP, left atrial pressure; PSM, propensity score matching; SpO_2_, oxygen saturation; TOF, tetralogy of Fallot.

This observational retrospective study was conducted at Fuwai Hospital, Beijing, China. The study was approved by the Institutional Review Committee, and the requirement for informed consent was waived. We collected the clinical data of 297 patients who underwent corrective operations for TOF (from October 2017 to December 2018). Key criteria for exclusion are as follows: (1) patients aged >6 years old; (2) preoperative use of mechanical ventilation, blood transfusion, and extracorporeal membrane oxygenation; (3) previous palliative surgery or complex abnormality; (4) emergency surgery; (5) left ventricular ejection fraction (LVEF) <50%; (6) incomplete postoperative LAP records as well as a postoperative LAP outside the normal range (6–12 mmHg); and (7) missing data for endpoint diagnosis. After applying the exclusion criteria, 140 patients were included in the analysis (Figure 1). In this study, patients were categorized into two groups according to their preoperative oxygen saturation (SpO_2_).

### Anesthetic and procedure

All patients underwent routine anesthetic management and CPB strategies at our institution. They were induced with midazolam (0.05–0.1 mg/kg), sufentanil (0.5–1 μg/kg), cisatracurium (0.2 mg/kg), and phencyclidine (0.02 mg/kg); anesthesia was maintained with inhaled sevoflurane, dexmedetomidine (1 μg/kg/h), cisatracurium (0.2–0.3 mg/kg/h), and sufentanil (2–3 μg/kg). Static inflation with airway pressure at 3–5 cm H_2_O was applied during CPB. SpO_2_, ECG, invasive blood pressure (radial artery), temperature (both nasopharynx and rectum), central venous pressure (5.5 F triple-lumen catheter into the right jugular vein), and LAP (20G single-lumen catheter into the right jugular vein and then into the left atrium after CPB through the atrium septum) were monitored routinely (7).

All patients were admitted to the ICU after TOF surgery. Inotropes were administrated by an intensivist. Patients were routinely treated with dopamine and dobutamine (3–8 μg/kg/min) and milrinone (0.2–1 μg/kg/min). Epinephrine and pituitrin were given as needed. Extubation was performed when patients met the following conditions: spontaneous respiration, hemodynamic stabilization, intact airway reflexes, manageable airway secretions, and chest x-ray excluding pulmonary edema and other lung diseases. The intensivist was responsible for the final decision to extubate or reintubate.

### Data collection and outcomes

Patient data related to the procedure and anesthetic were obtained from the medical records and checked by two independent medical researchers. The demographic data included age, sex, height, and weight. The body surface area (BSA) was calculated using height and weight (BSA = 0.0061 × height[cm] + 0.0128 × weight[kg] − 0.1529). Preoperative data included the diagnosis, SpO_2_, LVEF, interventricular septal thickness (IVST), left ventricular end-diastolic volume (LVEDV), and right and left pulmonary artery diameters. Operative data included the operation time, CPB time, aortic cross-clamp (ACC) time, minimum temperature during CPB, intraoperative blood loss, and ultrafiltration volume. The left ventricular end-diastolic volume index (LVEDVI) was calculated using LVEDV and BSA (LVEDVI=LVEDV/BSA), and the Nakata index was calculated using the PA area and BSA [Nakataindex=(rightPAarea+leftPAarea)/BSA]. Postoperative data included LAP 12 h after the operation, vasoactive-inotropic score (VIS) 12 h after the operation, intake and output volume during the day of surgery, LVEF on the day of surgery, and duration of MV before the first postoperative extubation and reintubation. One of the authors had full access to all the data in this study and took responsibility for their integrity and analysis.

Our study endpoint was prolonged MV, including failure to extubate within the first 48 h after the operation and reintubation. The postoperative LAP was the average value and was recorded as an integer.

### Statistical analysis

Statistical analysis was performed using Stata software version 17, and *P* < 0.05 was considered statistically significant. The participants were first stratified into SpO_2_ < 90% and SpO_2_ ≥ 90% groups. Propensity score matching (PSM) was used to balance the potential factors affecting the outcome. To create propensity score-matched pairs, we performed one-to-one matching using the Stata module of psmatch2 (8), in which the control variables in our study were used (LVEDVI, Nakata index, CPB time, and VIS) (9–12). Based on nearest neighbor matching, the 63 SpO_2_ < 90% cases were matched with 63 SpO_2_ ≥ 90% cases. Standardized differences were used as indicators of intergroup balance. If the standardized differences were less than 10.0%, the covariates between the two groups were considered well-balanced (13).

For summary statistics, categorical variables were described as numbers and percentages and non-normally distributed continuous variables were described as median and interquartile ranges. The Mann–Whitney *U*-test and chi-square test were performed to detect differences in outcomes between groups. Subgroup analysis was conducted to investigate the association between postoperative LAP and the occurrence of prolonged MV stratified by SpO_2_. LAP was categorized based on the cutoff points calculated using receiver-operating characteristic curves. The optimal cutoff point corresponded to the maximum value of the Youden index. The Youden index was calculated by sensitivity and specificity (Youdenindex=sensitivity+specificity−1) (14). Finally, the postoperative LAP threshold was obtained by calculating the between-group variation stratified by each level of LAP within the normal range.

## Results

### Sample characteristics of SpO_2_ < 90% and SpO_2_ ≥ 90% groups

A total of 140 patients (54.3% male) were included in this study. The mean age of the patients undergoing corrective surgery for TOF was 12.5 months. Some baseline characteristics showed statistically significant differences between the two groups before PSM. Higher CPB time (112 min vs. 95 min) and ACC time (76 min vs. 66 min) were observed in the SpO_2_ < 90% group. However, compared with patients in the SpO_2_ ≥ 90% group, patients in the SpO_2_ < 90% group showed a lower Nakata index (155.1 vs. 181.5) and LVEDVI (51.1 vs. 54.7). Using one-to-one PSM, 63 cases with SpO_2_ < 90% cases were matched with 63 cases with SpO_2_ ≥ 90%. The standardized percentage bias of covariates was close to 10% after PSM, showing a good match. Table 1 shows the demographic and clinical characteristics of the SpO_2_ < 90% and SpO_2_ ≥ 90% groups before and after PSM. The demographic and outcome-related characteristics of the SpO_2_ < 90% and SpO_2 _≥ 90% groups were no longer statistically different.

**Table 1:** Demographic and clinical data of TOF patients.

	Before PSM			After PSM		
	SpO_2 _< 90% (*n* = 72)	SpO_2 _≥ 90% (*n* = 68)	*P* value	SpO_2 _< 90% (*n* = 63)	SpO_2 _≥ 90% (*n* = 63)	*P* value
Demographics						
Age, months	8.9 (7–13)	9.6 (6.9–15.4)	0.980	8.8 (7–12.9)	10.5 (7.9–17.5)	0.193
Sex (male/female)	38/34	38/30	0.712	33/30	27/36	0.285
Height, cm	70 (66–74)	70 (65–75)	0.864	69 (66,73)	72 (65,75)	0.095
Weight, kg	9 (7,10)	9 (7–10)	0.801	9 (7–10)	9 (8–10)	0.376
Preoperative data						
HR, min^−1^	130 (120–132)	128 (120–136)	0.797	130 (120–134)	125 (120–140)	0.456
LVEF, %	69 (65–71)	66 (64–71)	0.099	69 (65–72)	66 (65–71)	0.318
IVST, mm	5 (4–5)	5 (4–5)	0.444	5 (4–5)	5 (4–5)	0.518
LVEDVI	51.1 (44.8–55.6)	54.7 (47.5–63.5)	0.011	52.30 (46.66–55.78)	51.30 (43.85–55.72)	0.566
Nakata index	155.1 (135.1–189.5)	181.5 (140.3–246.9)	0.023	165.2 (136.2–197.6)	158.9 (131.2–207.7)	0.571
SpO_2_, %	81 (70–85)	95 (92–98)	<0.001	82 (70–85)	92 (92–96)	<0.001
Intraoperative data						
Temperature min, °C	30 (28–30)	30 (28–30)	0.927	30 (28–30)	30 (28–30)	0.716
Time of operation, h	3 (3–4)	3 (3–4)	0.292	3 (3–4)	4 (3–4)	0.838
CPB time, min	112 (93–135)	95 (74–124)	0.008	110 (93–130)	108 (82–124)	0.211
ACC time, min	76 (62–100)	66 (51–84)	0.015	75 (62–99)	77 (51–90)	0.373
Intraoperative blood loss, ml	30 (20–30)	20 (20–30)	0.277	30 (20–30)	20 (20–30)	0.245
Ultrafiltration volume, ml	400 (295–500)	400 (300–543)	0.513	375 (295–485)	320 (250–600)	0.957
Postoperative data (12 h after operation)						
LAP, mmHg	7 (7–8)	7 (6–8)	0.890	7 (6–8)	7 (7–7)	0.435
LVEF, %	60 (59–64)	60 (60–65)	0.068	60 (60–64)	60 (60–65)	0.136
Fluid balance, ml/kg	181 (101.8–262.4)	194.1 (109.0–287.7)	0.400	186.3 (108–262.8)	223.2 (108.5–310.1)	0.056
VIS	10 (8–15)	10 (8–14)	0.516	10 (8–14)	10 (8–15)	0.953
MV, h	22 (16–51)	17 (9–27)	0.021	22 (15–48)	16 (7–25)	0.003

Data are expressed as the number (percentage) of patients or median (interquartile range). LAP is measured as the average value 12 h after operation. MV is defined as the duration of mechanical ventilation before the first postoperative extubation.

ACC, aortic cross-clamp; CPB, cardiopulmonary bypass; HR, heart rate; IVST, interventricular septal thickness; LAP, left atrial pressure; LVEDVI, left ventricular end-diastolic volume index; LVEF, left ventricular ejection fraction; MV, mechanical ventilation; SpO_2_, oxygen saturation; temperature min, minimum temperature; VIS, vasoactive-inotropic score.

### Hypoxia and prolonged mechanical ventilation

Table 2 shows the frequency of prolonged mechanical ventilation-associated events in the SpO_2_ < 90% and SpO_2_ ≥ 90% groups. The comparison between the two groups revealed a significant difference in failure to extubate within the first 48 h (*x*^2^ = 4.63, *P* = 0.031) and prolonged MV (*x*^2^ = 5.15, *P* = 0.023). The occurrence of failure to extubate within the first 48 h (23.81% vs. 9.52%) and prolonged MV (26.98% vs. 11.11%) was more commonly observed in children with preoperative SpO_2_ < 90%.

**Table 2:** Frequency of prolonged mechanical ventilation-associated events.

Prolonged mechanical ventilation	SpO_2 _< 90% (*n* = 63)	SpO_2 _≥ 90% (*n* = 63)	*P* value
Failure to extubate within the first 48 h	15 (23.81%)	6 (9.52%)	0.031
Reintubation	2 (3.17%)	2 (3.17%)	1.000
Total	17 (26.98%)	7 (11.11%)	0.023

In the SpO_2 _≥ 90% group, one patient meets failure to extubate within the first 48 h and reintubation at the same time.

SpO_2_, oxygen saturation.

### LAP and prolonged mechanical ventilation

**Figure 2:**
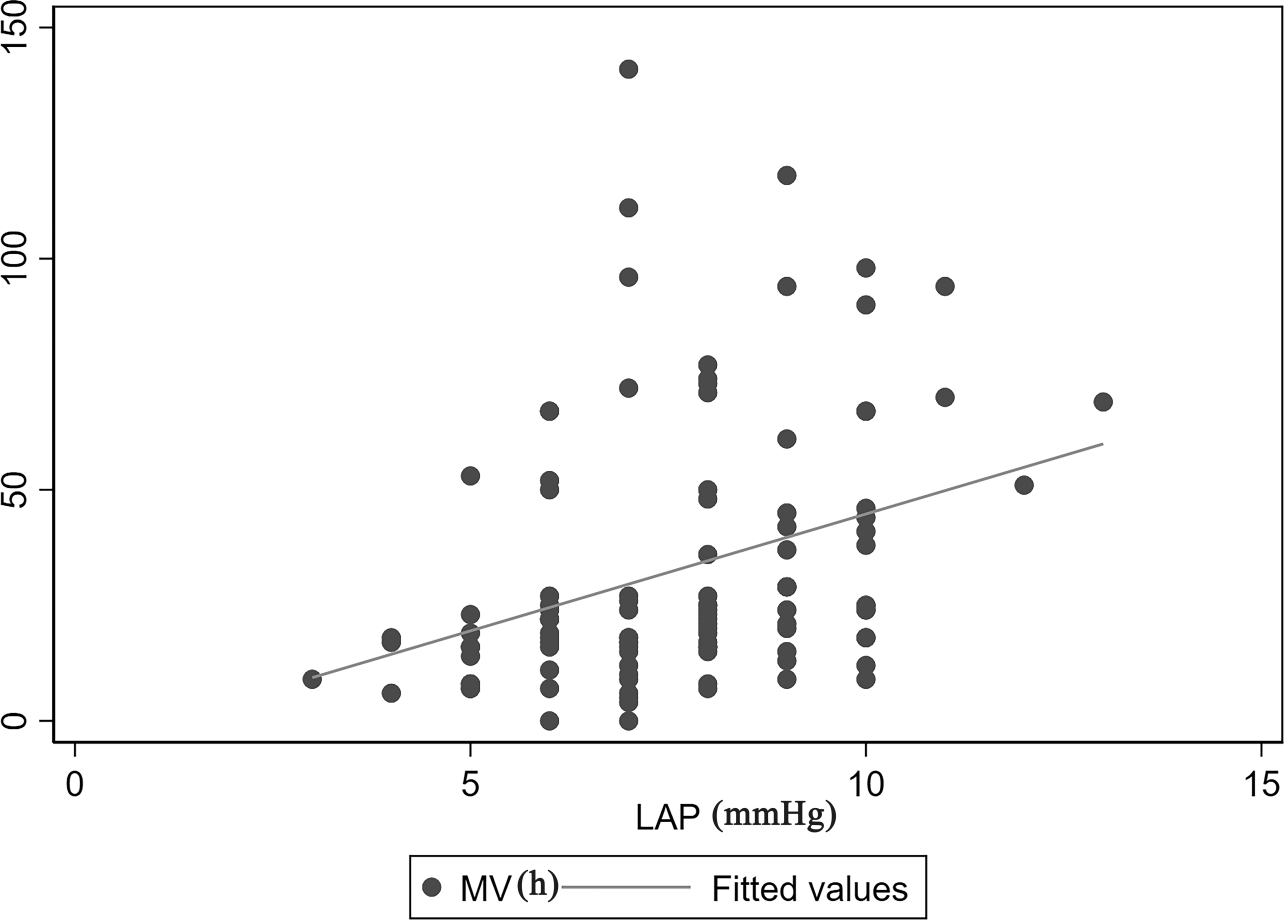
Correlation between MV time and LAP. There was a positive linear correlation between MV time and LAP (Spearman correlation coefficient = 0.41, *P* < 0.01). LAP, left atrial pressure; MV, mechanical ventilation.

**Figure 3:**
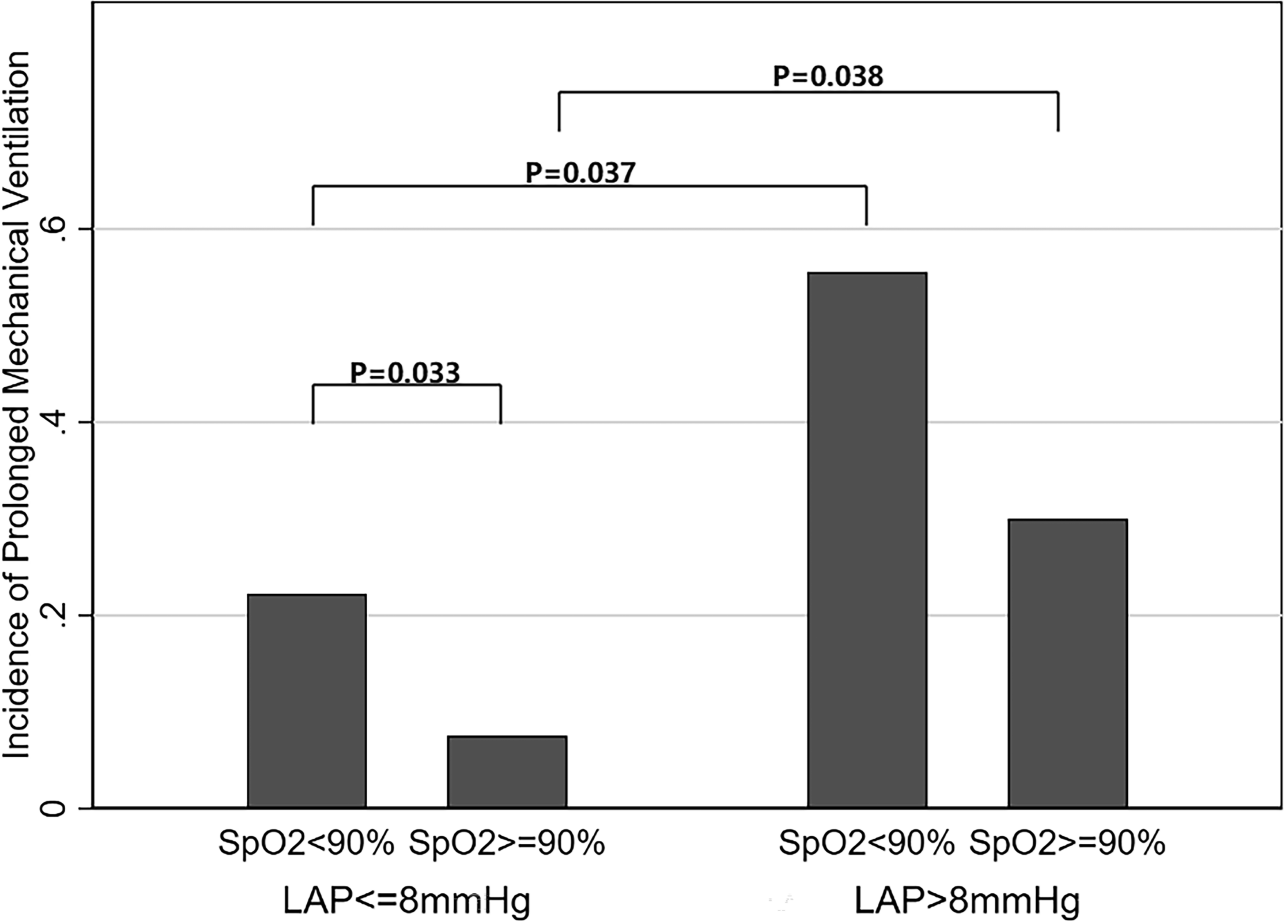
Relationship between LAP within the normal range (6–12 mmHg) and incidence of prolonged mechanical ventilation. The incidence of prolonged MV consistently increased with LAP in both the SpO_2_ < 90% (22.22% vs. 55.56%, *P* = 0.037) and SpO_2_ ≥ 90% (7.55% vs. 30.00%, *P* = 0.038) groups. Compared to children in the SpO_2_ ≥ 90% group, a higher occurrence of prolonged MV was observed in children with SpO_2_ < 90% (22.22% vs. 7.55%, *P* = 0.033). LAP, left atrial pressure; SpO_2_, oxygen saturation.

The correlation between MV time and LAP is demonstrated in Figure 2. There was a positive linear correlation between MV time and LAP (Spearman correlation coefficient = 0.41, *P* < 0.01). In addition, Figure 3 shows the association between LAP and prolonged MV stratified by SpO_2_. LAP was categorized based on the cutoff points (8 mmHg) calculated using receiver-operating characteristic curves. We found that the incidence of prolonged MV consistently increased with LAP in both the SpO_2_ < 90% (22.22% vs. 55.56%, *P* = 0.037) and SpO_2_ ≥ 90% group (7.55% vs. 30.00%, *P* = 0.038), although LAP was still within the normal range (6–12 mmHg). Compared to children in the SpO_2_ ≥ 90% group, a higher occurrence of prolonged MV was observed in children with SpO_2_ < 90% (22.22% vs. 7.55%, *P* = 0.033).

### Postoperative LAP threshold

We further compared the variation in prolonged MV between the SpO_2_ < 90% and SpO_2_ ≥ 90% groups based on each level of LAP within the normal range (Table 3). When LAP was ≥8 mmHg, a higher occurrence of prolonged MV was observed in the SpO_2_ < 90% group, with a statistically significant difference. In addition, no significant difference in the incidence of prolonged MV was found between the groups when the LAP was ≤7 mmHg. In other words, if we control LAP to ≤7 mmHg, the occurrence of prolonged MV in children under chronic hypoxia will be close to that in non-hypoxic children.

**Table 3:** Comparison of prolonged mechanical ventilation between groups based on each level of LAP within the normal range.

LAP (mmHg)	SpO_2 _< 90%	SpO_2 _≥ 90%	*P* value
6	2 (12.50%)	0 (0.00%)	0.171
≤7	7 (18.42%)	4 (8.16%)	0.153
≤8	12 (22.22%)	4 (7.55%)	0.033
≤9	14 (23.73%)	4 (7.14%)	0.014
≤10	15 (24.59%)	5 (8.33%)	0.016
≤11	15 (24.59%)	7 (11.11%)	0.050
≤12	17 (26.98%)	7 (11.11%)	0.023

LAP, left atrial pressure; SpO_2_, oxygen saturation.

## Discussion

In this study, we grouped patients based on preoperative SpO_2_ and regarded TOF patients with preoperative SpO_2_ < 90% as patients with chronic hypoxia (15). By measuring the analysis results above, we found that children with chronic hypoxic conditions were prone to a higher incidence of prolonged MV after cardiac surgery. Furthermore, we discussed the association between postoperative LAP and prolonged MV based on the premise that there were no remarkable differences in postoperative intake and output volume, LVEF, and VIS between the two groups. For pediatric patients with TOF, increasing LAP within the normal range has a substantial association with a high incidence of prolonged MV, and those in chronic hypoxic conditions tolerated lower postoperative LAP (≤7 mmHg) well.

TOF is a cyanotic congenital cardiac defect that was described as having four classical pathological processes: (1) pulmonary outflow tract obstruction, (2) ventricular septal defect, (3) overriding aortic root, and (4) right ventricular hypertrophy (16, 17). The development of the pulmonary vascular bed is strongly dependent on the flow. Pulmonary outflow tract obstruction results in reduced pulmonary blood flow, and the development and function of small pulmonary vessels are directly affected by diminished pulmonary flow in children with TOF (18). Postoperative fluid homeostasis in the lung depends on the integrity of the lung endothelial barrier (19). Breakdown of the barrier causes protein-rich pulmonary edema by increasing the flow of fluid and plasma proteins in the capillaries of the lungs (6). Based on the dysplasia of small pulmonary vessels in children with TOF, the fundamental reason for their susceptibility to postoperative lung exudation may be the disordered pulmonary vascular barrier function induced by chronic hypoxia and CPB surgery.

Many studies have established a paradigm in which hypoxia increases endothelial monolayer permeability (2–4). Other researchers reached the contradictory conclusion that pulmonary microvascular endothelial cells (PMVEC) grow to confluence under hypoxia, and incubation of endothelial monolayers under long-term hypoxic conditions forms a tighter and less permeable endothelial monolayer than similar cells grown under normoxia, as confirmed in both primary human and rat PMVEC (20). Chronic hypoxia before surgery may cause a number of compensatory changes in the lung tissue, particularly remodeling and hyperplasia of the pulmonary vessels (21). Furthermore, children undergoing surgical repair of complex congenital heart diseases tend to require a longer duration of CPB. It was reported that CPB increases pulmonary microvascular permeability and endothelial dysfunction through the Src kinase pathway and degradation of endothelial glycocalyx (5, 22). Therefore, pulmonary vascular barriers in cyanotic patients were weaker and more likely to be damaged after CPB surgery than those in noncyanotic patients, which makes cyanotic patients more likely to experience postoperative pulmonary exudation.

Increasing alveolocapillary barrier permeability or hydrostatic pressure gradients in the pulmonary circulation cause pulmonary edema to form (6). Studies have shown that increased pulmonary vascular pressure induces edema in animal models (23). In the absence of significant differences in postoperative cardiac function and volume, the LAP essentially represents the hydrostatic pressure of the pulmonary microcirculation. Elevated LAP leads to more fluid transfer to the capillary interstitial and alveolar spaces. Meanwhile, alveolar fluid reabsorption is impaired in the presence of elevated LAP (24) because a higher hydrostatic pulmonary circulation pressure inhibits active Na^+^ transport (25), which can result in more edema formation and pulmonary damage. It has also been reported that in rats with high LAP levels, lung permeability to large and small solutes increases progressively (25). High capillary pressures cause barrier damage, increasing permeability and fluid entry into the interstitium and alveoli. The underlying mechanism might be related to disruption of the endothelial junctional barrier by pressure-induced activation of Piezo1 (6). Accordingly, LAP is closely associated with pulmonary exudation and edema.

In cardiogenic pulmonary edema, excessive hydrostatic pressure across the pulmonary circulation causes swelling. For patients with a fragile pulmonary vascular barrier in chronic hypoxic conditions, it is beneficial to control hydrostatic pressure at a low state to maintain adequate tissue perfusion. LAP is affected by cardiac function, volume, and vasoactive drug intervention, reflecting the left ventricular preload when mitral valve function is normal. Thus, maintaining cardiac function, managing volume, and fluid balance, and monitoring the type and amount of vasoactive drugs are recommended to keep the LAP low. Even so, a very low level of LAP (≤5 mmHg) (26) leads to impairment in lung parenchymal mechanics because the absence of venous pressure may result in an unstable geometry of the alveolar space and deterioration of parenchymal mechanics (27).

Our research was done as rationally as possible. Covariates were selected as potential confounders based on previous studies. The variables associated with MV were age, LVEDVI, Nakata index, previous palliative operations, cardiac dysfunction, emergency status, transfusion, CPB time, and VIS (9, 11). Our exclusion criteria for the study design eliminated the impact of age, previous palliative operations, cardiac dysfunction, emergency status, and transfusion on the endpoint. In addition, we chose LVEDVI, Nakata index, CPB time, and VIS as adjusted covariates in the PSM. In our study, no differences were found in postoperative cardiac function and volume. Therefore, it is rational to discuss the influence of SpO_2_ and LAP on the study endpoint. This study is the first to provide the scope of postoperative LAP management for children with chronic hypoxia, which has great clinical significance in improving prognosis.

Nevertheless, this study has some limitations. First, our sample size was small; therefore, there was an obvious tendency for the incidence of prolonged MV between the SpO_2 _< 90% and SpO_2 _≥ 90% groups, but no statistically significant variation was found when the postoperative LAP was >8 mmHg. Second, it is unfortunate that not all adjusted variables’ standardized percentage bias was less than 10% after PSM. However, this did not affect baseline consistency between the two groups. The pseudo-*R*^2^, an indicator of overall covariate imbalance, was much lower after matching than it was before (after; pseudo-*R*^2^ = 0.004, mean bias = 5.9, median bias = 4.4, *P* = 0.949 vs. before; pseudo-*R*^2^ = 0.070, mean bias = 33.8, median bias = 35.7, *P* = 0.011). These findings suggest an adequate balance of covariate distribution among the matched groups. Third, this was a single-center study, which may have introduced selection bias. Thus, the results may not have a strong ability to be generalized. In the future, performing multicenter prospective studies will help validate our findings.

In conclusion, more importance should be given to pediatric heart disease patients with chronic hypoxic conditions before surgery. Children with chronic preoperative hypoxia were prone to a high occurrence of prolonged mechanical ventilation after correction of TOF. They did not tolerate higher postoperative LAP well. To improve pulmonary prognosis, it is better to control the postoperative LAP at a lower state level (≤7 mmHg).

## Data Availability

The datasets generated for this study will not be made publicly available because of the data protection policy in our hospital. Requests to access the datasets should be directed to the corresponding author.
